# Does maternal education moderate the relationship between adolescent cannabis use and mental health in early adulthood?

**DOI:** 10.1111/dar.13945

**Published:** 2024-09-09

**Authors:** Gemma Sawyer, Laura D. Howe, Matthew Hickman, Stanley Zammit, Lindsey A. Hines

**Affiliations:** 1 MRC Integrative Epidemiology Unit, Bristol Medical School, University of Bristol, Bristol, UK; 2 Population Health Sciences, Bristol Medical School, University of Bristol, Bristol, UK; 3 Medical Research Council Centre for Neuropsychiatric Genetics and Genomics, School of Medicine, Cardiff University, Cardiff, UK; 4 Department of Psychology, University of Bath, Bath, UK

**Keywords:** ALSPAC, cannabis, mental health, socioeconomic position

## Abstract

**Introduction:**

Socioeconomic disadvantage has been associated with cannabis use and poor mental health. It is therefore hypothesised that lower maternal education, a proxy for socioeconomic disadvantage, may increase the risk of cannabis-related mental health and substance use consequences.

**Methods:**

A total of 5099 participants from the Avon Longitudinal Study of Parents and Children reported cannabis use via questionnaires at 16 or 18. Logistic regression was used to examine the relationship between any and regular (weekly or more) adolescent cannabis use with depression, anxiety, psychotic experiences, and problematic cannabis use at age 24. Maternal education was included as an effect modifier. Missing data were addressed through multiple imputation using chained equations.

**Results:**

In total, 36.5% of participants reported adolescent cannabis use and, of these, 14% reported regular use. Adolescent cannabis use was associated with an increased likelihood of anxiety and problematic cannabis use; however, there was little evidence of moderation by maternal education. Regular cannabis use was associated with an increased likelihood of problematic cannabis use, with little evidence of moderation by maternal education. There was weak evidence that the association between regular cannabis use and depression (interaction *p*-value = 0.024) and anxiety (interaction *p*-value = 0.056) was stronger in people with high maternal education.

**Discussion and Conclusions:**

Adolescent cannabis use is associated with increased risk of anxiety and cannabis use disorder, but there was insufficient evidence that childhood socioeconomic position (proxied by maternal education) modifies this relationship. Improved public health messages for all adolescents about these risks may be warranted.

## 1 Introduction

Cannabis is the most commonly used illicit drug world-wide [1]. Adolescent cannabis use is associated with mental health disorders, including psychosis [2], depression, and anxiety [1], particularly with earlier and more frequent cannabis use [2]. Understanding this relationship is challenging due to complex and overlapping risk factors, such as lower socioeconomic position (SEP) [3]. SEP may confound the relationship between substance use and mental health, or it could moderate the relationship through a clustering of risk factors that impact resilience to substance effects.

Understanding the joint effects of cannabis and SEP on mental health may help inform targeted public health campaigns and interventions. Putative moderating effects of various SEP aspects, including education and income, have been observed for alcohol-related mortality, and for tobacco use and depression [4, 5], though have not been robustly replicated. Studies of moderation by SEP for cannabis-related outcomes are scarce [5] and weakened by cross-sectional design and focus on adults who tend to experience lesser harms than adolescents [2]. Moreover, there is need for further evidence to confirm that frequent cannabis use increases the risk of adverse consequences [1, 2], particularly regarding mental health consequences of adolescent use.

The current research aims to address gaps in existing literature by investigating the effect of adolescent cannabis use (and regular use) on adult mental health in a UK birth cohort, where cannabis is illegal. We also aim to explore whether this relationship is moderated, on a multiplicative scale, by maternal education as a proxy of childhood SEP using prospective, longitudinal data to minimise recall bias and reverse causality. We used maternal education as our proxy of childhood SEP because it captures knowledge-related assets and correlated aspects, such as income and occupation, and has been linked with offspring health [6]. Moreover, maternal education is available for the largest number of participants and is not influenced by changes to mother’s labour market participation during early years of parenting.

## 2 Methods

### 2.1 Study population

The Avon Longitudinal Study of Parents and Children (ALSPAC) is a UK population-based birth cohort which recruited pregnant women residing in the former Avon region with expected delivery dates between 1 April 1991 and 31 December 1992. Full details have been previously outlined [7–9]. The study website contains details of data available through a data dictionary and variable search tool (http://www.bristol.ac.uk/alspac/researchers/our-data/). Study data were collected and managed using Research Electronic Data Capture (REDCap) tools hosted at the University of Bristol. REDCap is a secure, web-based software platform designed to support data capture for research studies [10]. Ethical approval for the study was obtained from the ALSPAC Ethics and Law Committee and the Local Research Ethics Committees. Informed consent for the questionnaire and clinic data was obtained from participants following recommendations at the time.

**Figure 1:**
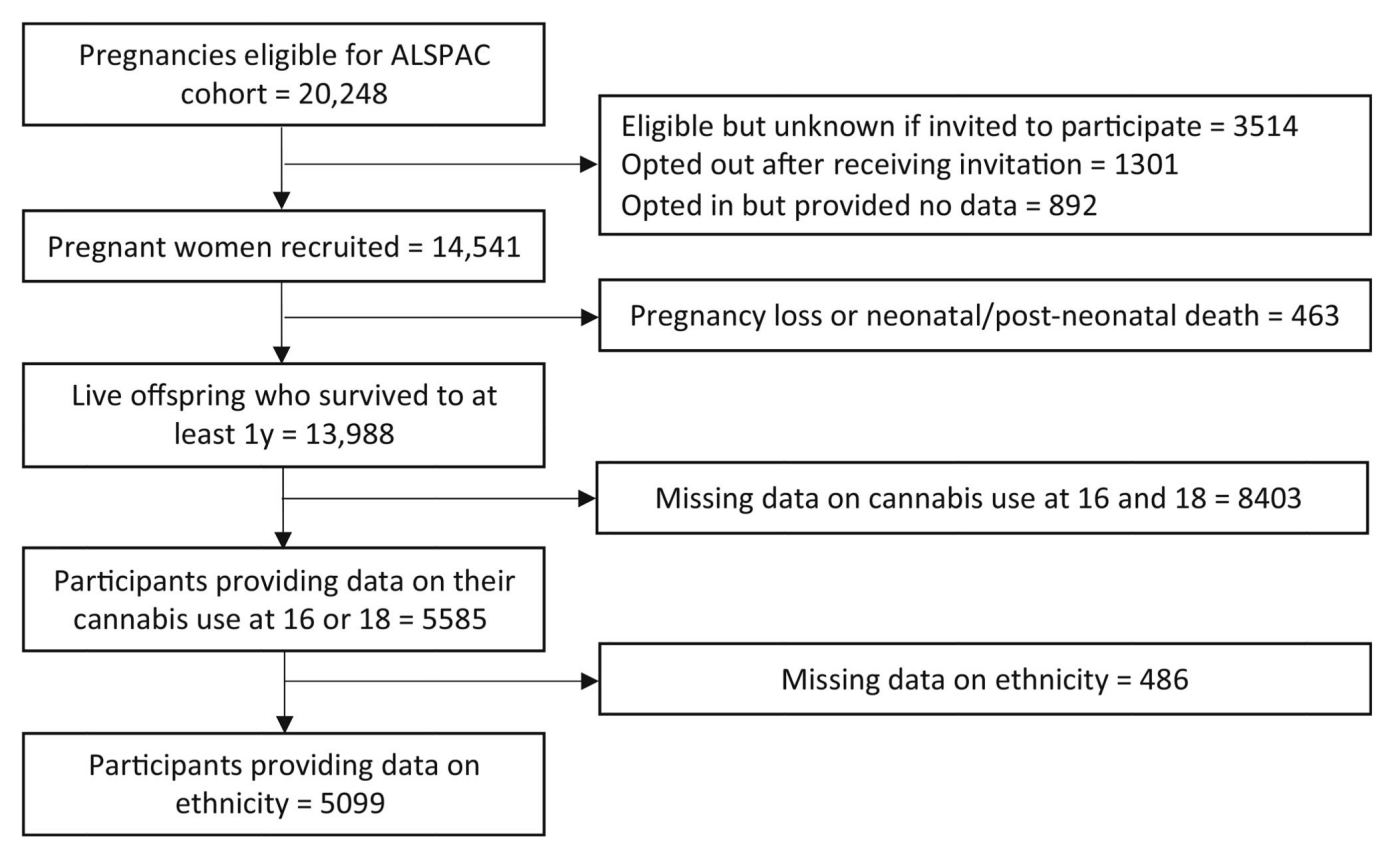
Flow diagram of the Avon Longitudinal Study of Parents and Children participants into this study sample.

Of the 14,541 pregnancies enrolled, 13,988 children were alive at age 1. A total of 5099 participants reported on cannabis use at 16/18 and a subsample of 1859 who reported using cannabis were eligible for analysis of cannabis use frequency (Figure 1).

### 2.2 Measures

#### 2.2.1 Exposures

At 16 and 18, participants were asked “Have you ever tried cannabis?” Individuals who reported ever using cannabis at 16 or 18 are classified as experiencing adolescent cannabis use. Those who reported cannabis use were asked to report frequency of use; this was dichotomised to occasional (monthly or less) and regular (weekly or more) (Supporting information S1).

#### 2.2.2 Outcomes

At 24, moderate/severe depressive episodes or generalised anxiety disorder (past week) were assessed with the Clinical Interview Schedule Revised and clinically significant criteria were applied [11]. Psychotic experiences (PE) were assessed with the Psychotic-Like Symptoms interview [12]. Participants were classified as having PEs if they reported a suspected/definite PE in the past 12 months, which was frequent or distressing. Problematic cannabis use in the past 6 months was measured with the Cannabis Abuse Screening Test (CAST; Supporting information S1) [13]. Responses were dichotomised to no/non-problematic (<2 CAST items) and problematic cannabis use (≥2 CAST items).

#### 2.2.3 Moderator

As a proxy for SEP, mothers self-reported their highest level of education during pregnancy, which we dichotomised to O (Ordinary) level (compulsory qualifications at 16) or below and A (Advanced) level (post-16 qualifications) or above.

#### 2.2.4 Confounders

Mother-reported confounders were parity, age at participant birth, and tobacco use during pregnancy. Participant confounders were sex assigned at birth, ethnicity, intelligence quotient at 8, depressive symptoms and PEs at 13, and tobacco and alcohol use at 16 (Supporting information S1).

### 2.3 Statistical analysis

Analyses were conducted in Stata, version 16. Logistic regression was used to estimate the association between maternal education and cannabis use at 16/18, and between cannabis use and mental health at 24, adjusting for confounders, followed by multivariable regression with maternal education as an effect modifier. Odds ratios (OR) and 95% confidence intervals (CI) are presented. This analysis was not pre-registered and should be considered exploratory.

### 2.4 Missing data

The sample was limited to those who had complete data on ethnicity and cannabis use at 16 or 18 (*N* = 5099). Missing data in outcomes and covariates were addressed through multiple imputation using chained equations (Supporting information S1).

## 3 Results

Of 5099 participants, 36.46% reported cannabis use at 16/18 and, of these, 14% reported using cannabis regularly. Table 1 presents the proportions of outcomes and covariates according to cannabis use.

### 3.1 Maternal education and cannabis use

Lower maternal education was associated with reduced odds of adolescent cannabis use (OR 0.81; 95% CI 0.72–0.91; Table 2), but there was little evidence of an association with regular use (OR 1.15; 95% CI 0.87–1.51).

### 3.2 Mental health and substance use

Those who reported adolescent cannabis use had increased odds of adulthood anxiety (OR 1.70; 95% CI 1.26–2.29) and problematic cannabis use (OR 3.17; 95% CI 1.40–7.17) after adjusting for confounders. Adolescent cannabis was not associated with depression (OR 1.30; 95% CI 0.89–1.88) or PEs (OR 1.16; 95% CI 0.77–1.74) following adjustment (Table 3).

Amongst those who used cannabis, regular use was associated with an 11-fold increase in the odds of problematic cannabis use (OR 11.30; 95% CI 5.92–21.57) at 24, after confounder adjustment. The association between regular cannabis and PEs was attenuated after adjusting for confounders (OR 1.80; 95% CI 0.94–3.44). There was little evidence regular cannabis use was associated with depression (OR 1.03; 95% CI 0.53–2.00) or anxiety (OR 1.11; 95% CI 0.65–1.90) at 24 (Table 4).

### 3.3 Effect modification by maternal education

For adolescent cannabis use, there was no evidence for effect modification, but estimates were slightly higher for low maternal education groups for depression (interaction *p* = 0.594), anxiety (interaction *p* = 0.240), PEs (interaction *p* = 0.994) and problematic cannabis use (interaction *p* = 0.809) at 24 (Table 3).

For frequent cannabis use, there was no evidence for effect modification for problematic cannabis use (interaction *p* = 0.729) or PEs (interaction *p* = 0.196), although the effect estimates were slightly higher for low maternal education groups (Table 4). Individuals with higher maternal education were slightly more likely to experience depression (interaction *p* = 0.024) and anxiety (interaction *p* = 0.056) following frequent cannabis use after adjustment for confounders.

The complete case results are in Tables S3–S6.

## 4 Discussion

This study found people with lower childhood SEP (proxied by maternal education) are less likely to use cannabis in adolescence, but no evidence that frequency of use differs. Adolescent cannabis use was associated with increased odds of anxiety, and both any and regular cannabis use were associated with increased odds of problematic use in adulthood. There was little statistical evidence of effect modification under a multiplicative model, meaning the relative odds of mental health outcomes in cannabis users or regular users compared with non-users or occasional users was similar regardless of maternal education, as a proxy of SEP.

This study builds upon previous literature reporting relationships between adolescent cannabis use and mental health by suggesting they may be accounted for by confounders [1, 14], notably earlier mental health, for which measures are often absent [1]. Previous work from this cohort found the cross-sectional relationship between higher-potency cannabis and PEs to be robust to adjustment for earlier PEs [15], whereas the present work indicates that longitudinal relationships are less robust to this adjustment. Future research would benefit from considering the temporality in onset of cannabis use and mental health.

To our knowledge, this is the first study examining the moderating effect of SEP on the relationship between adolescent cannabis use and later mental health. Our results support previous findings that low SEP groups are less likely to experiment with cannabis but more likely to use cannabis daily [16], as well as associations between cannabis and adverse mental health [1, 2]. When exploring regular cannabis use, there was weak evidence that the odds of depression and anxiety were greater in groups with high, compared with low, maternal education; however, the imprecise effect estimates make it difficult to identify the effect of cannabis in each group. This contrasts evidence relating to the relationship between SEP and mental health outcomes when cannabis exposure is not incorporated [17], and warrants exploration if robustly replicated. Otherwise, there was little evidence that SEP moderated the associations on a multiplicative scale, which is consistent with patterns of risk when there is co-exposure to cannabis use for psychiatric outcomes [18].

### 4.1 Strengths and limitations

This study is strengthened by use of prospective longitudinal data and adjustment for many confounders; however, residual confounding cannot be excluded. The reliance on self-reported cannabis use may result in misclassification, particularly given its illegality. Attrition prior to 16/18 may have resulted in underrepresentation of low SEP groups. Such factors may impact the nature of the observed associations, meaning replication in more representative cohorts would be useful. Whilst maternal education is a viable proxy for SEP, it does not encapsulate all SEP aspects or SEP later in childhood/adolescence. Further research to address these limitations would enable more robust conclusions, which could better aid in the development of public health interventions. Finally, the interaction results should be considered cautiously due to difficult interpretation with regards to the choice of statistical model, inferences from findings, and frequent lack of robust replication.

## 5 Conclusion

The findings suggest that adolescent cannabis use increases the odds of anxiety and problematic cannabis use in early adulthood. There was little evidence of moderation by childhood SEP (proxied by maternal education), supporting the need for improved public health messages for all adolescents about the risks of cannabis use.

## Supplementary Material

Supplement

## Figures and Tables

**Table 1:** Distribution of outcomes at 24, maternal factors, participant demographics, and substance use factors according to cannabis use at 16 or 18.

Characteristic	Adolescent cannabis use (*N* = 5099)				Frequency of cannabis use (*N* = 1859)		
	Yes (36.46%)	No (63.54%)	*p*-value		Weekly or more (14%)	Monthly or less (86%)	*p*-value
Outcomes							
Depression (moderate or severe symptoms) at 24	10.38%	6.90%	<0.001		12.18%	10.34%	<0.001
Generalised anxiety disorder at 24	12.40%	8.27%	<0.001		13.37%	12.43%	<0.001
Psychotic-like experiences at 24	6.76%	4.68%	<0.001		11.68%	6.47%	<0.001
Problematic cannabis use at 24	7.13%	2.71%	<0.001		29.94%	3.39%	<0.001
Demographic variables							
Low maternal education	48.90%	54.10%	<0.001		51.90%	48.41%	<0.001
Maternal smoker during pregnancy	51.35%	37.17%	<0.001		64.20%	49.24%	<0.001
Maternal age, years (mean [SD])	29.64 (0.108)	29.23 (0.078)	NA		29.49 (0.326)	29.67 (0.114)	<0.001
Maternal parity (mean [(SD])	0.79 (0.021)	0.70 (0.015)	NA		0.87 (0.062)	0.78 (0.023)	<0.001
Male sex	39.05%	42.13%	<0.001		55.31%	36.41%	<0.001
Black or minority ethnic group	5.33%	3.33%	<0.001		5.47%	5.30%	0.471
Below average IQ at 8	11.74%	16.08%	<0.001		13.70%	11.63%	<0.001
Adolescent substance use							
Alcohol use at 16	99.28%	90.93%	<0.001		99.04%	99.44%	<0.001
Tobacco use at 16	83.20%	26.68%	<0.001		87.82%	82.23%	<0.001
Adolescent mental health							
MFQ Score at 13 (mean [SD])	5.77 (0.136)	4.54 (0.084)	NA		6.46 (0.386)	6.62 (0.133)	NA
No. of PEs at 13 (mean [SD])	0.20 (0.015)	0.14 (0.010)	NA		0.27 (0.049)	0.19 (0.017)	NA

*Note*: Adolescent cannabis use refers to any reported cannabis use at 16/18 compared with no reported use, and regular cannabis use refers to weekly or more use amongst those reporting adolescent cannabis use at 16/18 compared with occasional use (monthly or less). Lower maternal education refers to O (Ordinary) Level or below (up to compulsory qualifications at 16). A *p-*value determined by *X*^2^ test. Abbreviations: IQ, intelligence quotient; MFQ, mood and feelings questionnaire; NA, not applicable; PE, psychotic experiences; SD, standard deviation.

**Table 2:** Logistic regression analysis of the association between maternal education and cannabis use outcomes in adolescence.

	Higher maternal education				Lower maternal education		
	*N*	OR (95% CI)	*p*-value		*N*	OR (95% CI)	*p*-value
Adolescent cannabis use	2437	1 (ref)	**—**		2662	0.81 (0.72-0.91)	<0.001
Regular cannabis use	950	1 (ref)	**—**		909	1.15 (0.87-1.51)	0.316

*Note*: High maternal education refers to individuals whose mothers’ reported education of A (advanced) Level or above (further post-16 qualifications and degrees) and low maternal education refers to individuals whose mothers reported education of O (ordinary) Level or below (up to compulsory qualifications at 16) during pregnancy. Adolescent cannabis use refers to any reported cannabis use at 16/18 compared with no reported use, and regular cannabis use refers to weekly or more use amongst those reporting adolescent cannabis use at 16/18 compared with occasional use (monthly or less). Abbreviations: CI, confidence interval; OR, odds ratio.

**Table 3:** Logistic regression analysis of the association between adolescent cannabis use and mental health and substance use outcomes in adulthood, with crude and adjusted effect modification analysis by maternal education.

		Unstratified analysis		Effect modification		
Outcome		OR (95% CI)	*p*-value	OR for high maternal education (95% CI)	OR for low maternal education (95% CI)	*p*-value for interaction^a^
Depression (moderate or severe symptoms)^b^	Unadjusted	1.56 (1.17–2.08)	0.003	1.45 (0.98–2.17)	1.69 (1.15–2.48)	0.586
	Adjusted	1.30 (0.89–1.88)	0.173	1.19 (0.74–1.92)	1.38 (0.88–2.17)	0.594
Generalised anxiety disorder	Unadjusted	1.57 (1.23–2.00)	<0.001	1.37 (0.94–1.99)	1.79 (1.28–2.48)	0.300
	Adjusted	1.70 (1.26–2.29)	0.001	1.41 (0.94–2.12)	1.94 (1.32–2.85)	0.240
Psychotic-like experiences^c^	Unadjusted	1.48 (1.05–2.09)	0.027	1.43 (0.86–2.40)	1.57 (1.00–2.47)	0.790
	Adjusted	1.16 (0.77–1.74)	0.486	1.15 (0.66–2.02)	1.16 (0.69–1.93)	0.994
Problematic cannabis use	Unadjusted	2.91 (1.39–6.07)	0.005	2.89 (0.64–12.98)	3.36 (1.47–7.67)	0.863
	Adjusted	3.17 (1.40–7.17)	0.006	2.94 (0.64–13.51)	3.68 (1.36–9.96)	0.809

*Note*: High maternal education refers to individuals whose mothers’ reported education of A (advanced) Level or above (further post-16 qualifications and degrees) and low maternal education refers to individuals whose mothers’ reported education of O (ordinary) Level or below (up to compulsory qualifications at 16) during pregnancy. Adolescent cannabis use refers to any reported cannabis use at 16/18 compared with no reported use. Adjusted for sex, ethnicity, maternal age, maternal smoking, parity, intelligence quotient at age 8, tobacco and alcohol use at age 16. Abbreviations: CI, confidence interval; OR, odds ratio.

a Wald test of the interaction parameter.

b Depressive symptoms at age 13.

c Psychotic experiences at 13.

**Table 4:** Logistic regression analysis of the association between regular cannabis use and mental health and substance use outcomes in adulthood, with crude and adjusted effect modification analysis by maternal education.

		Unstratified analysis		Effect modification		
Outcome		OR (95% CI)	*p*-value	OR for high maternal education^a^ (95% CI)	OR for low maternal education^b^ (95% CI)	*p*-value for interaction^c^
Depression (moderate or severe symptoms)^d^	Unadjusted	1.19 (0.67–2.12)	0.548	1.90 (0.91–3.98)	0.73 (0.32–1.67)	0.079
	Adjusted	1.03 (0.53–2.00)	0.936	2.05 (0.90–4.66)	0.53 (0.21–1.35)	0.024
Generalised anxiety disorder	Unadjusted	1.08 (0.65–1.80)	0.768	1.76 (0.91–3.41)	0.65 (0.28–1.51)	0.070
	Adjusted	1.11 (0.65–1.90)	0.707	1.89 (0.96–3.72)	0.67 (0.28–1.58)	0.056
Psychotic-like experiences^e^	Unadjusted	1.89 (1.04–3.45)	0.037	1.15 (0.39–3.36)	2.36 (1.15–4.85)	0.265
	Adjusted	1.80 (0.94–3.44)	0.075	0.98 (0.30–3.15)	2.42 (1.13–5.18)	0.196
Problematic cannabis use^f^	Unadjusted	12.41 (6.55–23.49)	<0.001	12.37 (5.45–28.07)	12.91 (5.18–32.16)	0.948
	Adjusted	11.30 (5.92–21.57)	<0.001	9.89 (4.17–23.48)	12.50 (4.89–31.92)	0.729

*Note*: Adjusted for sex, ethnicity, maternal age, maternal smoking, parity, intelligence quotient at age 8, tobacco use at age 16. Abbreviations: CI, confidence interval; OR, odds ratio.

a High maternal education refers to individuals whose mothers’ reported education of A (advanced) Level or above (further post-16 qualifications and degrees).

b Low maternal education refers to individuals whose mothers’ reported education of O (ordinary) Level or below (up to compulsory qualifications at 16) during pregnancy.

c Wald test of the interaction parameter.

d Depressive symptoms at age 13.

e Psychotic experiences at 13.

f Regular cannabis use refers to weekly or more use amongst those reporting adolescent cannabis use at 16/18 compared with occasional use (monthly or less).

## Key Points

- Adolescent cannabis use was less likely amongst those with lower maternal education.
- Anxiety and problematic cannabis use were more likely in those who used cannabis in adolescence.
- Frequent cannabis use in adolescence was associated with a further increase in likelihood of problematic cannabis use in adulthood.
- There was little evidence that such relationships were altered by socioeconomic position (proxied by maternal education).
