# Supplementary material for: Does maternal education moderate the relationship between adolescent cannabis use and mental health in early adulthood?
Source: Drug Alcohol Rev. Author manuscript; Available in PMC 2024 Nov 15. (PMC7616805; doi:10.1111/dar.13945)
Supplement: Supplement [file EMS199898-supplement-Supplement.docx]

# SUPPORTING INFORMATION

## Supplementary methods

### Exposures

*Cannabis frequency.* At 16, participants were asked which of the following statements suited them best: “I have only ever tried cannabis once or twice”; “I used to sometimes use or take cannabis but I never do now”; “I sometimes use or take cannabis but less often than once a week”; “I usually use or take cannabis between once and six times a week”; “I usually use or take cannabis more than six times a week, but not every day”; or “I usually use or take cannabis every day.” At 18, participants were asked which of the following statements reflected their frequency of cannabis use in the past 12 months: “once or twice”; “less than monthly”; “monthly”; “weekly”; “daily”; or “almost daily”. Participants highest reported frequency at 16 or 18 was used.

*Problematic cannabis use.* There are six CAST items. At 24, participants were asked whether, in the past year, they had used cannabis before midday, they had used cannabis when they were alone, they had suffered any memory problems, friends and family had said they needed to reduce their cannabis consumption, they had unsuccessfully attempted to reduce or stop using cannabis, or they had experienced any problems due to their cannabis use. Reports of two or more CAST items were used to define problematic cannabis use [1].

### Confounders

We selected variables for inclusion as confounders if they were known to, or could plausibly, cause both exposure (cannabis use at 16/18) and outcome (mental health or problematic cannabis use in adulthood). Maternal confounders included self-reported parity (number of previous live births), age at delivery, and ever smoker status (ever or never) at 18 weeks’ gestation. Child confounders included sex and ethnicity (White or non-White), which were reported by mothers during pregnancy. IQ was measured at age 8 through a short form Weschler Intelligence Scale for Children [2] and responses were dichotomised to “Below Average” and “Average or Above”. Depressive symptoms at 13 were obtained through a short computer-based version of the Mood and Feelings Questionnaire (score ranging from 0 to 26; higher scores indicate more depressive symptoms) [3], and PEs (number of suspected or definite PEs) were assessed through an interview based on the Schedule for Affective Disorders and Schizophrenia for School-Age Children [4] and the Diagnostic Interview Schedule-Children [5]. Finally, to control for other adolescent substance use, participants were asked “Have you ever drunk alcohol?” (ever or never) and “Have you ever smoked a cigarette?” (ever or never) at 16.

### Missing data

Separate imputation models were run for the exposure of adolescent cannabis use (Table S1) and the exposure of cannabis use frequency (limited to a subsample who reported using cannabis at 16/18; Table S2). Imputation models were stratified by maternal education to allow for modelling of interactions in the analyses.

For the imputation of outcomes and covariates for the exposure of adolescent cannabis use, the exposure, outcomes and covariates were entered into imputation models, along with following auxiliary variables: cannabis use frequency at age 20, tobacco use at age 18, tobacco use at age 20, alcohol use at age 18, alcohol use at age 20, MFQ score at age 16, MFQ score at age 18, PLIKS score at age 17, CAST score at age 22, and IQ at age 15.

For the imputation of the cannabis frequency exposure, outcomes and covariates for the exposure of frequent cannabis use (a subsample limited to those who had report cannabis use at age 16/18), the exposure, outcomes and covariates were entered into imputation models, along with following auxiliary variables: cannabis use frequency at age 20, tobacco use at age 18, tobacco use at age 20, alcohol use at age 18, alcohol use at age 20, MFQ score at age 16, MFQ score at age 18, PLIKS score at age 17, CAST score at age 22, and IQ at age 15. The covariate of alcohol use at age 16 was not included in this model, or in analyses relating to this exposure, due to perfect prediction.

Estimates were obtained by pooling results across 40 imputed datasets using Rubin’s rules, accounting for the within and between imputation variance [6], and guided by results of Monte Carlo error tests, which assess statistical reproducibility of the imputation [7].

## Supplementary results

There was a low number of observations reporting each mental health outcome by adolescent cannabis use (Table S5) and frequency of cannabis use (Table S6) as stratified by maternal education. As there were no participants with high maternal education who reported regular adolescent cannabis use and psychotic experiences at age 24, this effect modification analysis could not be conducted.

## Supplementary references

1. Legleye S, Piontek D, Kraus L. Psychometric properties of the Cannabis Abuse Screening Test (CAST) in a French sample of adolescents. Drug Alcohol Depend. 2011;113:229–35.
2. Horwood J, Salvi G, Thomas K, Duffy L, Gunnell D, Hollis C, et al. IQ and non-clinical psychotic symptoms in 12-year-olds: Results from the ALSPAC birth cohort. Br J Psychiatry. 2008;193:185–91.
3. Costello EJ, Angold A. Scales to assess child and adolescent depression: checklists, screens, and nets. J Am Acad Child Adolesc Psychiatry. 1988;27:726–37.
4. Kaufman J, Birmaher B, Brent D, Rao U, Flynn C, Moreci P, et al. Schedule for affective disorders and schizophrenia for school-age children-present and lifetime version (K-SADS-PL): Initial reliability and validity data. J Am Acad Child Adolesc Psychiatry. 1997;36:980–8.
5. Shaffer D, Fisher P, Lucas CP, Dulcan MK, Schwab-stone ME. NIMH Diagnostic Interview Schedule for Children Version IV (NIMH DISC-IV): Description, differences from previous versions, and reliability of some common diagnoses. J Am Acad Child Adolesc Psychiatry. 2000;39:28-38.
6. Rubin D. Multiple Imputation for Nonresponse in Surveys [Internet]. Hoboken, NJ, USA: John Wiley & Sons, Inc; 1987 [cited 6 August 2022]. Available from: <https://onlinelibrary.wiley.com/doi/book/10.1002/9780470316696>
7. White IR, Royston P, Wood AM. Multiple imputation using chained equations: Issues and guidance for practice. Stat Med. 2011;30:377–99.

## Supplementary tables

Table S1. Proportion of missing data in the sample of individuals who provided data on their cannabis use age 16/18 (N=5099) and comparison of prevalence of outcomes and covariates in the incomplete and imputed samples

| **Role in analysis** | **Variable** | **Proportion missing data in sample restricted to those reporting on cannabis use age 16/18** | **Prevalence/mean in sample restricted to those reporting on cannabis use age 16/18** | **Prevalence/mean in imputed dataset** |
| --- | --- | --- | --- | --- |
| Outcomes | Depression at age 24 | 46.5% | 8.0% | 8.0% |
|  | Anxiety at age 24 | 46.6% | 9.6% | 9.8% |
|  | Psychotic experiences at age 24 | 47.6% | 5.4% | 5.4% |
|  | Problematic cannabis use at age 24 | 69.8% | 3.6% | 4.3% |
| Covariate | Low maternal education | 7.2% | 52.6% | 52.2% |
|  | Male sex | 0.2% | 40.4% | 41.0% |
|  | Non-White ethnicity | 8.4% | 4.1% | 4.1% |
|  | Tobacco | 9.6% | 46.7% | 47.3% |
|  | Alcohol | 9.6% | 93.6% | 94.0% |
|  | Maternal smoker | 6.3% | 42.7% | 42.3% |
|  | Low IQ at 8 | 22.4% | 13.9% | 14.4% |
|  | Maternal age, mean (SD) | 4.5% | 29.3 (4.6) | 29.4 |
|  | Parity, mean (SD) | 7.1% | 0.7 (0.9) | 0.7 |
|  | MFQ at 13, mean (SD) | 26.9% | 4.9 (4.5) | 5.0 |
|  | PEs at 13, mean (SD) | 21.3% | 0.2 (0.5) | 0.2 |

Abbreviations: IQ, intelligence quotient; MFQ, mood and feelings questionnaire; PEs, psychotic experiences. Lower maternal education refers to O (Ordinary) Level or below (up to compulsory qualifications at 16).

Table S2. Proportion of missing data in the sample of individuals who reported using cannabis age 16/18 and reported on their frequency of use (N=1859) and comparison of prevalence of outcomes and covariates in the incomplete and imputed samples

| **Role in analysis** | **Variable** | **Proportion missing data in sample restricted to those endorsing cannabis use age 16/18** | **Prevalence/mean in sample restricted to those endorsing cannabis use age 16/18** | **Prevalence/mean in imputed dataset** |
| --- | --- | --- | --- | --- |
| Outcomes | Depression at age 24 | 45.2% | 10.4% | 10.6% |
|  | Anxiety at age 24 | 45.4% | 12.6% | 12.6% |
|  | Psychotic experiences at age 24 | 46.4% | 7.0% | 7.2% |
|  | Problematic cannabis use at age 24 | 50.0% | 5.6% | 7.1% |
| Covariate | Low maternal education | 7.0% | 48.5% | 48.9% |
|  | Male sex | 0.1% | 38.3% | 39.1% |
|  | Non-White ethnicity | 8.6% | 5.4% | 5.3% |
|  | Tobacco | 9.9% | 84.5% | 83.0% |
|  | Maternal smoker | 6.3% | 48.1% | 51.3% |
|  | Low IQ at 8 | 19.7% | 10.8% | 11.9% |
|  | Maternal age, mean (SD) | 4.8% | 29.6 (4.8) | 29.6 |
|  | Parity, mean (SD) | 7.2% | 0.8 (0.9) | 0.8 |
|  | MFQ at 13, mean (SD) | 25.9% | 5.6 (4.8) | 5.7 |
|  | PEs at 13, mean (SD) | 19.7% | 0.2 (0.6) | 0.2 |

Abbreviations: IQ, intelligence quotient; MFQ, mood and feelings questionnaire; PEs, psychotic experiences. Lower maternal education refers to O (Ordinary) Level or below (up to compulsory qualifications at 16).

Table S3. Association between cannabis use at 16 or 18 and outcomes at 24, maternal factors, participant demographics, and substance use factors in the complete case sample (N=961).

| **Characteristic** | **Adolescent cannabis use (N= 961)** | | | **Frequency of cannabis use (N=554)** | | |
| --- | --- | --- | --- | --- | --- | --- |
|  | **Yes**  N= 554 (57.65%) | **No**  N= 407 (42.35%) | **P value** | **Weekly or more**  N= 71 (12.82%) | **Monthly or less**  N= 483 (87.18%) | **P value** |
| Outcomes | | | | | | |
| **Depression (moderate or severe symptoms) at 24** | 46 (8.3) | 31 (7.62) | 0.698 | 6 (8.45) | 40 (8.28) | 0.962 |
| **Generalised anxiety disorder at 24** | 55 (9.93) | 37 (9.09) | 0.663 | 10 (14.08) | 45 (9.32) | 0.210 |
| **Psychotic-like experiences at 24** | 27 (4.87) | 17 (4.18) | 0.610 | 5 (7.04) | 22 (4.55) | 0.363 |
| **Problematic cannabis use at 24** | 25 (4.51) | <5 | <0.001* | 15 (21.13) | 10 (2.07) | <0.001 |
| Demographic variables | | | | | | |
| **Low maternal education** | 202 (36.53) | 158 (38.82) | 0.468 | 33 (46.48) | 169 (35.06) | 0.062 |
| **Maternal smoker during pregnancy** | 250 (45.13) | 144 (35.38) | 0.002 | 44 (61.97) | 206 (42.65) | 0.002 |
| **Maternal age, years**  Mean (SD) | 30.72 (4.38) | 29.87 (4.15) | 0.002** | 30.89 (4.98) | 30.70 (4.29) | 0.734** |
| **Maternal parity**  Mean (SD) | 0.76 (0.89) | 0.67 (0.83) | 0.102** | 0.87 (0.94) | 0.74 (0.88) | 0.243** |
| **Male sex** | 210 (37.91) | 176 (43.24) | 0.095 | 38 (53.52) | 172 (35.61) | 0.004 |
| **Black or minority ethnic group** | 20 (3.61) | 13 (3.19) | 0.726 | <5 | 17 (3.52) | 0.732* |
| **Below average IQ at 8** | 26 (4.69) | 36 (8.85) | 0.010 | <5 | 23 (4.76) | 0.842 |
| Adolescent substance use | | | | | | |
| **Alcohol use at 16** | 551 (99.46) | 395 (97.05) | 0.003 | 70 (98.59) | 481 (99.59) | 0.286 |
| **Tobacco use at 16** | 450 (81.23) | 145 (35.63) | <0.001 | 104 (18.77) | 262 (64.37) | <0.001 |
| Adolescent mental health | | | | | | |
| **MFQ score at 13**  Mean (SD) | 5.72 (4.64) | 4.47 (4.24) | <0.001* | 6.30 (5.39) | 5.64 (4.52) | 0.267** |
| **No. of PEs at 13**  Mean (SD) | 0.19 (0.62) | 0.19 (0.56) | 0.956** | 0.27 (0.70) | 0.18 (0.61) | 0.269** |

Abbreviations: IQ, intelligence quotient; MFQ, mood and feelings questionnaire; PEs, psychotic experiences; SD, standard deviation. Lower maternal education refers to O (Ordinary) Level or below (up to compulsory qualifications at 16). P value determined by X^2^ test or Fisher’s exact* for binary variables and t-test** for continuous variables. Frequencies less than five are reported <5 to adhere to ALSPAC’s disclosure rules.

Table S4. Logistic regression analysis of the association between low maternal education and cannabis use outcomes in adolescence in the complete case sample (N=961).

|  | **Higher maternal education** | | | **Lower maternal education** | | |
| --- | --- | --- | --- | --- | --- | --- |
|  | **N** | **OR** (95% CI) | **P value** | **N** | **OR** (95% CI) | **P value** |
| **Adolescent cannabis use** | 601 | 1 (ref) | – | 360 | 0.91 (0.70-1.18) | 0.468 |
| **Regular cannabis use** | 352 | 1 (ref) | – | 202 | 1.61 (0.97-2.66) | 0.064 |

Abbreviations: CI, confidence interval; OR, odds ratio. High maternal education refers to individuals whose mothers’ reported education of A (advanced) Level or above (further post-16 qualifications and degrees) and low maternal education refers to individuals whose mothers’ reported education of O (ordinary) Level or below (up to compulsory qualifications at 16) during pregnancy. Adolescent cannabis use refers to any reported cannabis use at 16/18 compared to no reported use, and regular cannabis use refers to weekly or more use amongst those reporting adolescent cannabis use at 16/18 compared to occasional use (monthly or less).

Table S5. Logistic regression analysis of the association between adolescent cannabis use and mental health and substance use outcomes in adulthood, with crude and adjusted effect modification analysis by maternal education in the complete case sample (N=961).

| **Outcome** | | **Unstratified analysis** | | **Effect modification** | | |
| --- | --- | --- | --- | --- | --- | --- |
|  |  | **OR** (95% CI) | **P value** | **OR for high maternal education** (95% CI) | **OR for low maternal education** (95% CI) | **P value for interaction*** |
| **Depression (moderate or severe symptoms)**^a^ | Unadjusted | 1.10 (0.68-1.77) | 0.699 | 1.12 (0.58-2.14) | 1.11 (0.55-2.22) | 0.984 |
|  | Adjusted | 0.95 (0.55-1.63) | 0.849 | 0.95 (0.47-1.92) | 0.95 (0.44-2.04) | 0.993 |
| **Generalised anxiety disorder** | Unadjusted | 1.10 (0.71-1.71) | 0.663 | 1.09 (0.60-1.99) | 1.15 (0.61-2.18) | 0.907 |
|  | Adjusted | 1.07 (0.65-1.78) | 0.783 | 0.99 (0.51-1.90) | 1.19 (0.59-2.39) | 0.687 |
| **Psychotic-like experiences**^b^ | Unadjusted | 1.18 (0.63-2.19) | 0.610 | 1.81 (0.69-4.73) | 0.84 (0.36-1.97) | 0.244 |
|  | Adjusted | 1.00 (0.49-2.05) | 0.993 | 1.60 (0.57-4.48) | 0.65 (0.25-1.69) | 0.380 |
| **Problematic cannabis use** | Unadjusted | 9.57 (2.25-40.64) | 0.002 | 8.78 (1.30-67.95) | 10.80 (1.40-83.46) | 0.888 |
|  | Adjusted | 15.10 (1.91-119.42) | 0.010 | 11.55 (1.09-122.60) | 21.65 (1.22-383.41) | 0.694 |

Abbreviations: CI, confidence interval; OR, odds ratio. High maternal education refers to individuals whose mothers’ reported education of A (advanced) Level or above (further post-16 qualifications and degrees) and low maternal education refers to individuals whose mothers’ reported education of O (ordinary) Level or below (up to compulsory qualifications at 16) during pregnancy. Adolescent cannabis use refers to any reported cannabis use at 16/18 compared to no reported use. Adjusted for maternal age, maternal smoking, parity, IQ at age 8, tobacco and alcohol use at age 16, and depressive symptoms at age 13^a^ or psychotic experiences at age 13^b^. *Wald test of the interaction parameter.

Table S6. Logistic regression analysis of the association between regular cannabis use and mental health and substance use outcomes in adulthood, with crude and adjusted effect modification analysis by maternal education in the complete case sample (N=554).

| **Outcome** | | **Unstratified analysis** | | **Effect modification** | | |
| --- | --- | --- | --- | --- | --- | --- |
|  |  | **OR** (95% CI) | **P value** | **OR for high maternal education** (95% CI) | **OR for low maternal education** (95% CI) | **P value for interaction*** |
| **Depression (moderate or severe symptoms)**^a^ | Unadjusted | 1.02 (0.42-2.51) | 0.962 | 1.13 (0.32-3.98) | 0.84 (0.23-3.03) | 0.742 |
|  | Adjusted | 0.93 (0.36-2.41) | 0.882 | 1.43 (0.38-5.33) | 0.65 (0.17-2.48) | 0.409 |
| **Generalised anxiety disorder** | Unadjusted | 1.60 (0.76-3.33) | 0.213 | 1.82 (0.65-5.10) | 1.26 (0.44-3.62) | 0.622 |
|  | Adjusted | 1.45 (0.67-3.13) | 0.348 | 1.79 (0.61-5.21) | 1.19 (0.40-3.50) | 0.596 |
| **Psychotic-like experiences**^b^ | Unadjusted | 1.59 (0.58-4.34) | 0.367 | NA | NA | NA |
|  | Adjusted | 1.44 (0.49-4.23) | 0.509 | NA | NA | NA |
| **Problematic cannabis use** | Unadjusted | 12.67 (5.43-29.55) | <0.001 | 9.59 (2.92-31.49) | 15.47 (4.42-54.16) | 0.588 |
|  | Adjusted | 9.60 (3.96-23.28) | <0.001 | 6.13 (1.76-21.39) | 15.17 (4.12-55.80) | 0.326 |

Abbreviations: CI, confidence interval; NA, not applicable; OR, odds ratio. High maternal education refers to individuals whose mothers’ reported education of A (advanced) Level or above (further post-16 qualifications and degrees) and low maternal education refers to individuals whose mothers’ reported education of O (ordinary) Level or below (up to compulsory qualifications at 16) during pregnancy. Regular cannabis use refers to weekly or more use amongst those reporting adolescent cannabis use at 16/18 compared to occasional use (monthly or less). Psychotic-like experiences effect modification analysis was not able to be conducted due to the sample size being too low. Adjusted for maternal age, maternal smoking, parity, IQ at age 8, tobacco use at age 16 and depressive symptoms at age 13^a^. *Wald test of the interaction parameter.
